# Improved usability of a multi-infusion setup using a centralized control interface: A task-based usability test

**DOI:** 10.1371/journal.pone.0183104

**Published:** 2017-08-11

**Authors:** Frank Doesburg, Fokie Cnossen, Willem Dieperink, Wouter Bult, Anne Marie de Smet, Daan J. Touw, Maarten W. Nijsten

**Affiliations:** 1 University of Groningen, University Medical Center Groningen, Department of Critical Care, Groningen, The Netherlands; 2 University of Groningen, Institute of Artificial Intelligence and Cognitive Engineering, Groningen, The Netherlands; 3 University of Groningen, University Medical Center Groningen, Department of Clinical Pharmacy and Pharmacology, Groningen, The Netherlands; 4 University of Groningen, University Medical Center Groningen, Department of Pharmacokinetics, Toxicology and Targeting, Groningen, The Netherlands; University Medical Center Utrecht, NETHERLANDS

## Abstract

The objective of this study was to assess the usability benefits of adding a bedside central control interface that controls all intravenous (IV) infusion pumps compared to the conventional individual control of multiple infusion pumps. Eighteen dedicated ICU nurses volunteered in a between-subjects task-based usability test. A newly developed central control interface was compared to conventional control of multiple infusion pumps in a simulated ICU setting. Task execution time, clicks, errors and questionnaire responses were evaluated. Overall the central control interface outperformed the conventional control in terms of fewer user actions (40±3 vs. 73±20 clicks, p<0.001) and fewer user errors (1±1 vs. 3±2 errors, p<0.05), with no difference in task execution times (421±108 vs. 406±119 seconds, not significant). Questionnaires indicated a significant preference for the central control interface. Despite being novice users of the central control interface, ICU nurses displayed improved performance with the central control interface compared to the conventional interface they were familiar with. We conclude that the new user interface has an overall better usability than the conventional interface.

## Introduction

In the last decades human factors research has been acknowledged as crucial in the development of high risk medical equipment [1–3]. Infusion pumps are among the most frequently used medical devices, and are used for the controlled intravenous (IV) administration of many infusion fluids and drug solutions. Erroneous use of infusion pumps may lead to (temporary) under- or overdosing of vital drugs, with potentially severe consequences. The usability of infusion pumps has often been identified as an important contributor to the incidence of such medication errors [4–8]. Various studies using heuristic analysis have already pointed to various design flaws in pumps [7,9,10]. Factors such as inadequate training, workflow interruptions or high workload can further increase the likelihood of such errors [7,11,12]. In some studies where new user interfaces were designed and tested, the focus was on the usability of individual pumps [13–15]. However, this is a situation not representative of a critical care setting where patients are often treated with over six pumps simultaneously. Also, medication errors are more frequent in the critical care arena than in any other hospital department, while patients who are most severely ill may be even more vulnerable to such errors [11,16–19]. Hence, in the current proof of concept study we focused on the IV delivery system as a whole instead of focusing on individual pumps.

We designed a new user interface for the centralized monitoring and control of multiple infusion pumps by ICU nurses (S1 Fig). Actions that would normally be performed directly on the infusion pump’s user interface, such as changing an infusion rate, can now be performed on a new central bedside user interface. The central user interface forwards the appropriate commands to each individual pump under its control. We hypothesized that such a more user-friendly interface would reduce the risk of user errors and would also improve the workflow and user satisfaction compared to the conventional control of multiple infusion pumps. The objective of this study was to develop and evaluate the possible usability benefits of a bedside central control interface for multiple infusion pumps compared to conventional multi-pump operation. For this purpose we followed the ISO 9241–11 definition of usability: “The extent to which a product can be used by specified users to achieve specified goals with effectiveness, efficiency and satisfaction in a specified context of use” [20].

## Materials and methods

### Hardware and connectivity

The availability of a relatively large touch screen (Samsung SM-T900; 12.2 inch screen diagonal) and the availability of a USB host mode were decisive in choosing the Android platform. The USB-serial-for-Android library, licensed under GNU Lesser General Public License (LGPL) Version 2.1, was used to facilitate serial communication between the Android tablet and the infusion pumps [21]. Three Alaris Asena GH Syringe pumps (Carefusion, United Kingdom) with firmware v2.3.6 were used. Physical connectivity was achieved using a Startech UUSBOTG micro-USB to USB OTG cable, a generic 4-port USB hub and three Startech ICUSB2321F USB-to-serial converters. Pump communication followed the Alaris Asena communication protocol [22]. The pumps were attached to a generic rack in a stacked fashion and a König & Meyer 19740 tablet clamp was used to hold the Android tablet in place.

A Windows laptop running a Java-based application was used to generate a quasi-randomized task order and display the current task.

In the central control condition, the Android tablet running a fully functional prototype of the central control interface was attached to the rack at shoulder height, facing the nurse (S1 Fig). In the conventional control condition, a Startech ICUSB2324X USB-to-RS232 converter was used to read all pump logs during the experiment.

In both conditions, the top pump was equipped with an empty syringe labeled as containing potassium chloride, the middle pump’s syringe was labeled with insulin, and the bottom pump’s syringe was labeled as containing propofol. Three other syringes with the same three labels were available for when a ‘change syringe’ task had to be performed.

### User interface

Development of the central control interface followed an iterative cycle where a design phase was alternated by review by ICU nurses. A think-aloud protocol (verbalization of thoughts) was used during these reviews to uncover possible weaknesses in the design [23]. Development was considered complete when the reviewing nurses and the development team required no further changes to the user interface. In the main user interface, pumps are represented by a single row on screen (S1 Fig). Each row or pump contains essential information, such as drug names, administration rates and concentrations. Buttons for basic pump functionalities, such as start/stop, administration rate, bolus, volume to be infused (VTBI) and advanced settings were also visible in the user interface. The settings menu included an option to reset the administered volume, pump connection details and a log of user actions. Pump alarms were cached by the central control interface and were highlighted in white text on a semi-transparent red overlay on top of the affected pump.

A new functionality not present in the conventional control interface (i.e. the individual pump interfaces) was an advanced VTBI menu which helped calculate the required administration rate based on a target dose or volume, the concentration of the drug on the pump and a predefined timeframe (S2 Fig). A change syringe menu displayed a checklist of all required steps, which upon completion of the steps would automatically restart the pump when the new syringe was correctly placed in the pump (S3 Fig). Additionally, the bolus menu allowed to set a predefined bolus volume which could be administered without the need for holding the bolus button throughout the entire procedure.

### Participants

Eighteen nurses from an adult ICU participated in the experiment. Their mean ± SD age was 41±12 years and their mean ± SD ICU experience was 12±12 years. Participants were randomly assigned to either the central control or the conventional control condition first. There were no significant differences in age or work experience between the groups. All participants volunteered for the experiment and had not been exposed to the central control interface before. Ethical approval for this study was waived by our institutional review board (M17.214943). There were no patients involved in this study and data was collected anonymously.

### Experimental tasks

Participants performed several typical tasks related to intravenous therapy in a simulated ICU setting. Examples of such tasks were changing administration rates, replacing syringes, administering boluses and navigating through menus. A between-subject design was used where participants performed the experiment using either conventional pump control or the centralized control interface. Each participant performed a set of thirteen pump-related tasks. Some tasks could only be performed when another was finished. For example, the volume to be infused (VTBI) functionality could only be turned off after it had been set up in the first place. However, such interdependent tasks did not necessarily follow each other directly; any number of other tasks could be scheduled in between. An overview of all experimental assignments is displayed in Table 1. Step-by-step workflows of each task type are included in S1 File. Note that some task types occurred more often than others in the experiment (change rate; replace syringe, setup and stop VTBI) in order to reflect their frequency in the real-world ICU environment.

**Table 1:** Description of experimental assignments.

Task	Description	Type
1	Stop the propofol 2% pump.	Stop pump
2	Restart the propofol 2% pump and change the administration rate to 3.5 ml/h. Note: this task requires task 1 to be completed first.	Restart & change rate
3	Change the administration rate of the potassium chloride pump to 2.4 ml/h.	Change rate
4	Change the administration rate of the potassium chloride pump to 3.0 ml/h.	Change rate
5	Change the administration rate of the insulin pump to 1.4 ml/h.	Change rate
6	Change the administration rate of the insulin pump to 0.8 ml/h.	Change rate
7	Administer a bolus of 3 ml propofol 2% at a rate of 500 ml/h.	Bolus
8	Replace the potassium chloride syringe and restart the pump.	Replace syringe
9	Replace the insulin syringe and restart the pump.	Replace syringe
10	Use the VTBI (volume to be infused) functionality to administer 3 mmol of potassium chloride using the current administration rate.	Setup VTBI
11	Use the volume to be infused (VTBI) functionality to administer 4 international units (IU) of insulin in 30 minutes. Make sure the pump stops after infusion.	Setup VTBI
12	Turn off the VTBI functionality on the potassium chloride pump, but make sure the pump keeps running. Note: this task requires task 10 to be completed first.	Stop VBTI
13	Turn off the VTBI functionality on the insulin pump, but make sure the pump keeps running. Note: this task requires task 11 to be completed first.	Stop VBTI

### Experimental procedure with the conventional and new central control interfaces

At the start of the experiment participants received a verbal explanation on all relevant functionalities of the user interface. Depending on the experimental condition either the central control interface or the conventional pump interface was explained. Participants had the opportunity to try out each interface before the start of the experiment. Experimental assignments were displayed one by one on a laptop with an application that was programmed for this purpose. Every participant had to complete the same set of assignments, although the assignment order was quasi-randomized. Participants were instructed to read and perform the task belonging to each assignment. After completion of the assignment the participant could click a “next assignment” button that would display the next assignment until all thirteen assignments were completed. After the completion of the tasks, a usability questionnaire was administered.

The usability questionnaire consisted of 19 5-point Likert scale statements about the user interface that the participant operated during the experiment. Participants used the scale to rate their agreement from 1 (lowest level of agreement) to 5 (highest level of agreement). Statements covered the overall system appearance, user experience and user interaction as well as the ease of use during the experimental assignments.

### Data collection & analysis

All user actions were logged during the course of the experiment. In order to assess usability of each user interface as defined in the ISO 9241–11 standard, we measured task execution times and clicks to reflect efficiency. More clicks indicated that more effort was required to perform a task. We deduced errors from experimental logs as a measure of effectiveness, and administered a questionnaire to measure user satisfaction [20]. We defined an error as any unintended deviation from achieving the intended outcome of an action that could not be attributed to an external influence [24]. Statistical differences for execution times, clicks and errors between conditions were analyzed with Student’s *t-*tests. Statistical differences in ratings on individual questionnaire statements were analyzed using the Mann-Whitney *U* test, and the mean questionnaire rating was analyzed with the Student’s *t*-test. All statistical tests were performed using IBM SPSS Statistics 22.

## Results

Table 2 displays the mean execution times, clicks and total number of errors per assignment type. The overall mean execution time (conventional vs. central mean ± SD, 406±119 vs. 421±108 seconds, not significant) did not differ between interfaces, whereas the overall mean number of clicks was lower using the central control interface (73±20 vs. 40±3 clicks, p < 0.001). Overall fewer errors were made using the central control interface (2.9±2.3 vs. 0.9±1.0 errors, p < 0.05). A description of all recorded errors is available in Table 1 in S1 File.

**Table 2:** Execution times, clicks and errors per assignment type.

Assignment type	Stop pump (task 1)		Restart & change rate (task 2)		Change rate (task 3–6)		Bolus (task 7)		Replace syringe (task 8 and 9)		Setup VTBI (task 10 and 11)		Cancel VTBI (task 12 and 13)		Overall mean (task 1–13)	
Interface	Conventional N = 9	Central N = 9	Conventional N = 9	Central N = 9	Conventional N = 9	Central N = 9	Conventional N = 9	Central N = 9	Conventional N = 9	Central N = 9	Conventional N = 8	Central N = 9	Conventional N = 8	Central N = 9	Conventional N = 9	Central N = 9
**Executiontime (s)**	18 ± 16	19 ± 16	13 ± 6*	26 ± 15*	27 ± 23	21 ± 10	79 ± 90	67 ± 25	20 ± 5**	50 ± 19**	63 ± 15*	46 ± 13*	22 ± 13	17 ± 6	406 ± 119	421 ± 108
**Clicks**	1.2 ± 0.7	1.0 ± 0.0	4.7 ± 3.5	2.8 ± 0.7	7.0 ± 1.8**	2.1 ± 0.2**	2.8 ± 1.6*	4.9 ± 2.2*	3.4 ± 0.6**	5.1 ± 0.9**	11.9 ± 4.5**	3.4 ± 0.5**	5.2 ± 3.2	3.0 ± 0.5	73 ± 20**	40 ± 3**
**Errors**	0.2 ± 0.6	0.0 ± 0.0	0.1 ± 0.3	0.0 ± 0.0	0.1 ± 0.2	0.0 ± 0.0	0.1 ± 0.3	0.3 ± 0.5	0.0 ± 0.0	0.1 ± 0.2	0.7 ± 0.5*	0.2 ± 0.4*	0.4 ± 0.5*	0.0 ± 0.0*	2.9 ± 2.3*	0.9 ± 1.0*

Data are expressed as mean ± standard deviation.

For errors, totals per category are displayed.

* = p < 0.05.

** = p < 0.001.

Median ratings on questionnaire statements and Mann-Whitney *U* test results can be found in Table 2 in S1 File. Ratings on statements regarding the aesthetics, clarity, intuitiveness and ease to discriminate between pumps with a user interface (statements 2–5) differed significantly between conditions in favor of the central control interface. The mean ± SD rating calculated over all questionnaire statements was higher for the central control interface than the conventional interface (4.6±0.3 vs. 4.1±0.5, p = 0.03).

## Discussion

We investigated whether a new central control interface would improve the overall usability of infusion pump control in a multi-infusion setting. Task-based usability analysis indicated that both objectively and subjectively the central control interface improved usability. Means calculated over all assignments indicated that participants required fewer clicks to perform the experimental tasks in the central control condition and also made fewer errors, indicating a more efficient and effective interaction. Questionnaires indicated that participants preferred the central control interface over the conventional interface, indicating greater user satisfaction.

There was no overall difference in execution times. This is remarkable since all participants were expert users of the conventional interface and had no prior experience with the central control interface. Moreover, it is likely that with further training the central control interface will outperform the conventional interface in terms of task execution times [25]. Data on specific execution times revealed that participants were able to change syringes 30 seconds faster on average using conventional pumps than using the central control interface. A difference between the two conditions is that the central control interface automatically stops and restarts the pump when a new syringe is placed. Although this feature was intended to improve the workflow, a relatively slow data connection between the central control interface and the infusion pumps limited the number of pump control commands to roughly 1 command per second. Hence, the pump’s RS232 communication layer was slowing down the task. As the communication protocol for the infusion pumps did not allow the automated confirmation of the syringe, the syringes had to be confirmed by manually pressing the confirm button on the pump instead of on the central control interface, which also impaired workflow. In case of a follow up of this study, a low latency communication layer between the pumps and interface will be required to optimize the task of replacing syringes.

In setting up a volume to be infused (VTBI) infusion on the pump participants were faster in the central control condition. The central control interface automatically calculated the required administration rate based on a preconfigured drug concentration, a target dose or volume and a predefined timeframe. In the conventional condition, the pumps offered no calculation support in the VTBI menu. Therefore, participants had to calculate which rate was required, which is a slow and error-prone task. Roughly half of the participants indicated that they did not regularly use the VTBI menu in the conventional interface, which may also explain that significantly more errors occurred during the VTBI tasks using the conventional interface. The difference in overall error rate appears to be mostly driven by errors in the VTBI tasks. The participants mentioned that the menu structure of the conventional interface was too complicated for the VTBI tasks. We believe this illustrates how counterintuitive design choices for the VTBI menu have contributed to poor usability and the occurrence of errors. This issue may be specific to the brand and model of infusion pump used in this study. A revision of the workflow of this particular VTBI menu should be considered if this proves to be an issue in real hospital environments as well.

In this study we compared the usability of a newly developed central control interface to that of a conventional pump setup. The results of this study indicate that this central control interface has a better overall usability than the conventional interface. However, this does not imply that central control in general will have a better usability than a conventional setup of individual pumps. In order to test for such a difference the layout and workflow of the central control interface should mimic that of the separate pumps. Such a setup will have very limited added value for a central control interface as it does not add any new features to the system as a whole. Added value may be gained by implementing sophisticated multi-pump profiles, for example, a system that switches from one pump to another when a syringe is almost empty or more complex multi-drug profiles (e.g. oncology treatment) [26]. Another extension could be early detection of IV line occlusions using combined pressure sensor readings from multiple pumps, which may reduce alarm fatigue by reducing the number of false alarms [27]. We believe that a well-designed central interface is complementary to a set of well-designed individual infusion pumps in a multi-infusion setting.

As a future extension of this study we propose testing with larger number of participants as well as ensuring a low-latency connection with the pumps. Testing with nurses who are not familiar with the conventional interface may reveal performance differences in other tasks than just the VTBI tasks. Including a longer training period with the central control interface may reveal differences in execution times as well. Although the current study did not focus on alarms, a future challenge for central pump control will be the channeling of alarm signals as existing visual and audible cues will remain important for the swift localization of the affected pump.

## Conclusion

In this proof of concept study, we have shown how the usability of infusion pumps can affect the occurrence of errors related to intravenous therapy. A user-friendly user interface to control and monitor multiple infusion pumps was developed and its usability was compared to that of the current method of manually operating multiple infusion pumps in a task-based usability analysis. Results suggest that the centralized control system has an overall better usability and reduces the number of errors.

## Supporting information

S1 Fig The user interface in the central control condition. (TIF)

S2 Fig Dialog of the volume to be infused menu of the central user interface. An infusion rate can be automatically calculated based on a desired volume and time window to avoid calculation errors.
(TIF)

S3 Fig Change syringe dialog. The change syringe dialog in the central user interface allows the user to check subtasks when they are done. The system communicates with the pump in the background to verify completion of these subtasks and will automatically restart the infusion in that case.
(TIF)

S1 File Supplementary material. (DOCX)

S1 Dataset Experimental data per category. (SAV)

S2 Dataset Experimental data. (SAV)

S3 Dataset Questionnaire data. (SAV)
