# Supplementary figures and images for: Improved usability of a multi-infusion setup using a centralized control interface: A task-based usability test
Source: PLoS One. 2017 Aug 11;12(8):e0183104. doi: 10.1371/journal.pone.0183104 (PMC5553772; doi:10.1371/journal.pone.0183104)

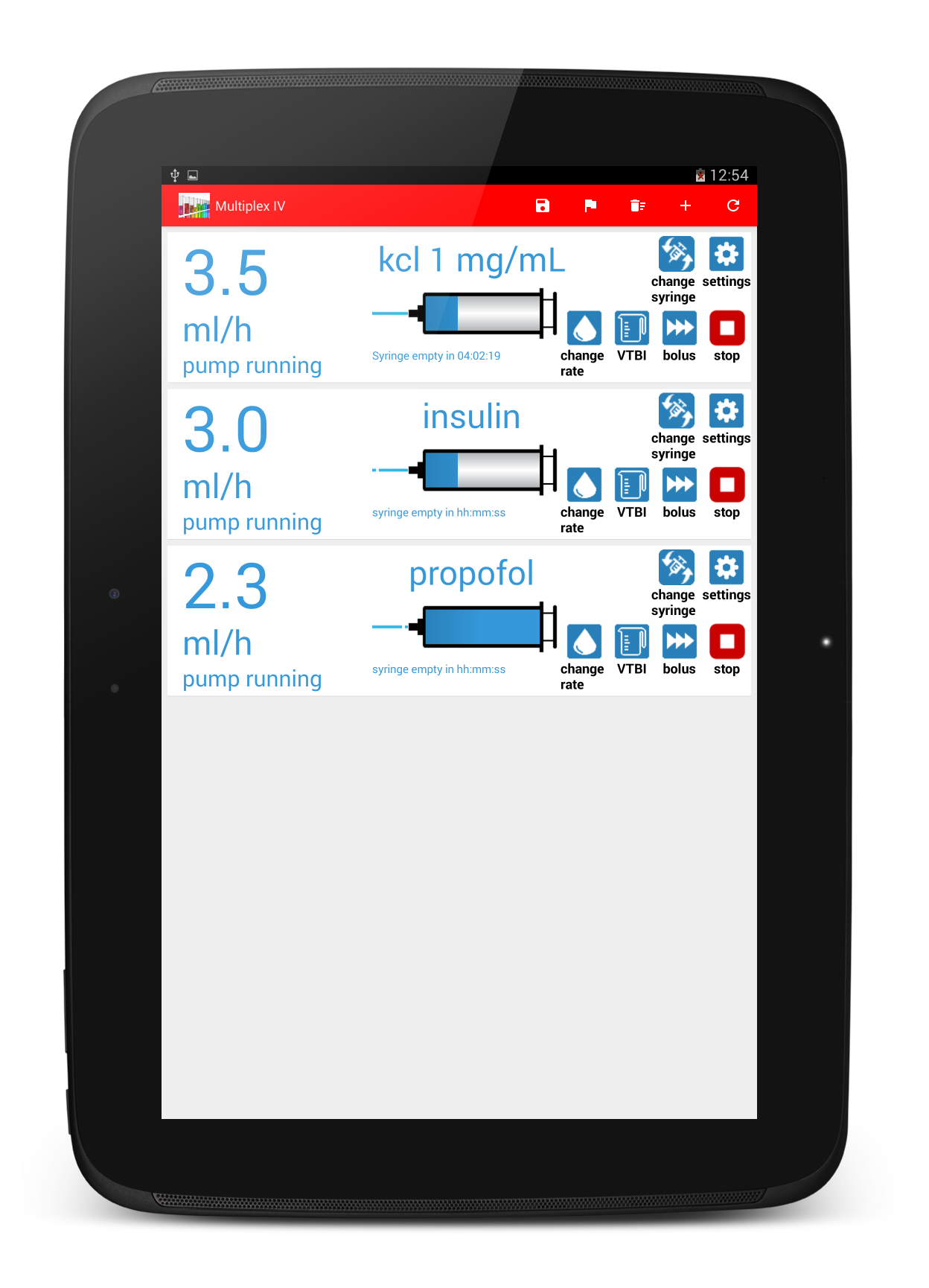

Supplement: S1 Fig — (TIF) [file pone.0183104.s001.tif]

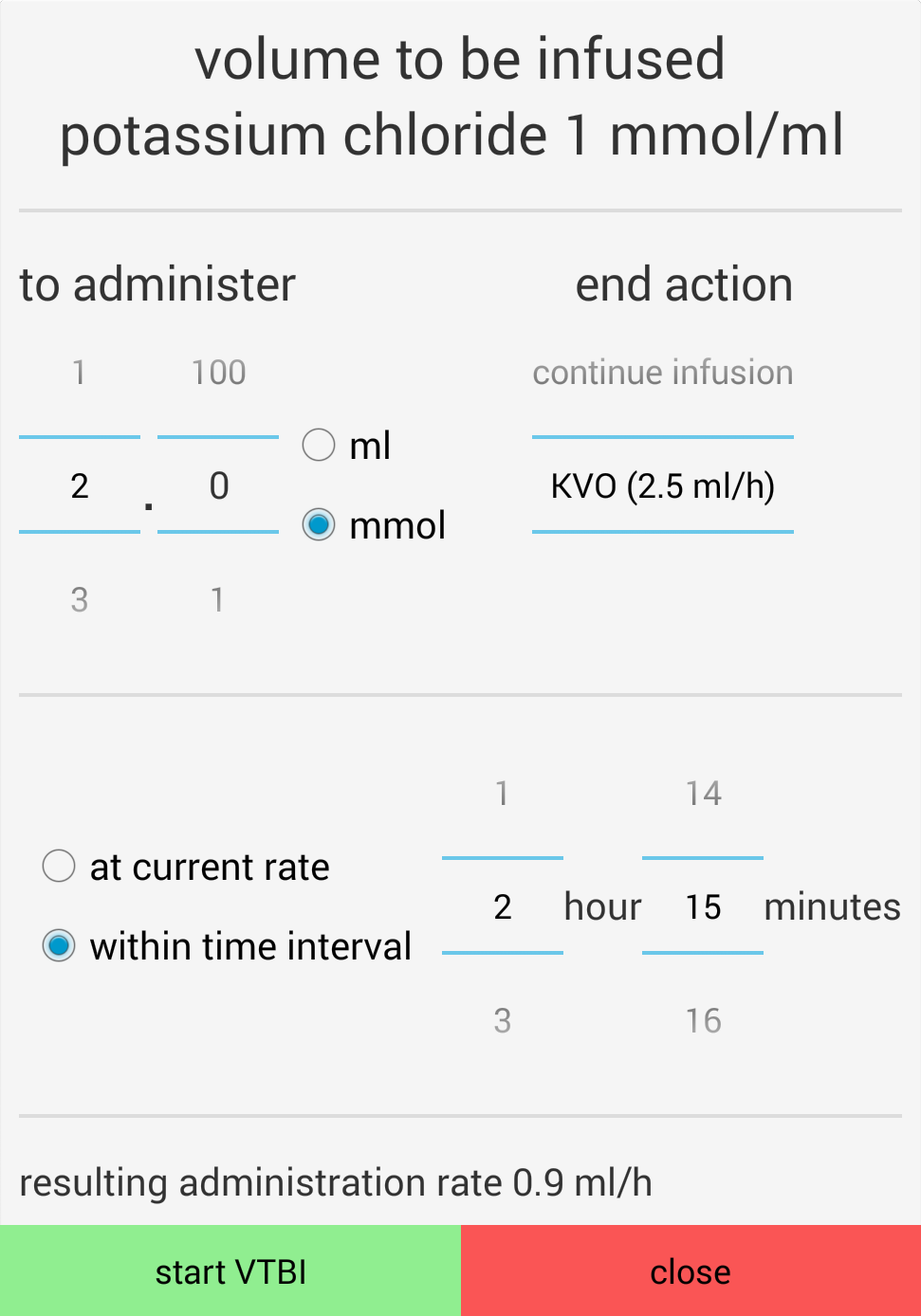

Supplement: S2 Fig — An infusion rate can be automatically calculated based on a desired volume and time window to avoid calculation errors. (TIF) [file pone.0183104.s002.tif]

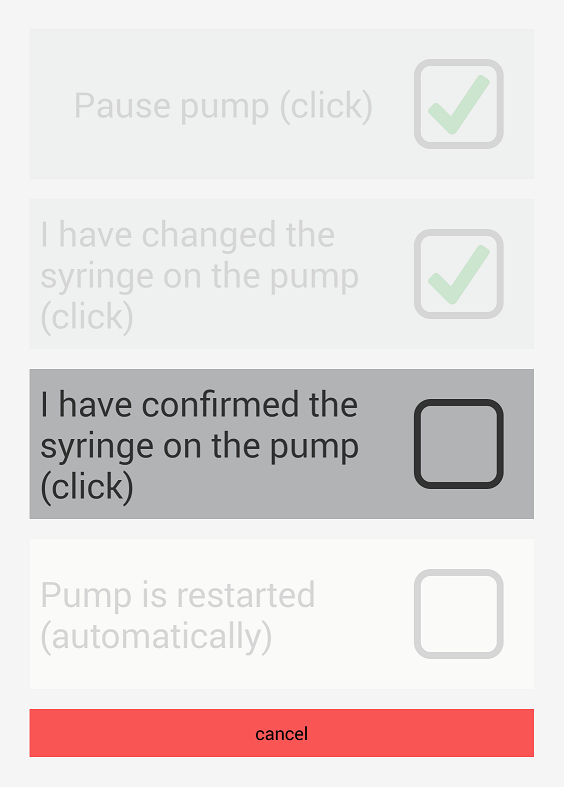

Supplement: S3 Fig — The change syringe dialog in the central user interface allows the user to check subtasks when they are done. The system communicates with the pump in the background to verify completion of these subtasks and will automatically restart the infusion in that case. (TIF) [file pone.0183104.s003.tif]
