# Supplementary material for: Improved usability of a multi-infusion setup using a centralized control interface: A task-based usability test
Source: PLoS One. 2017 Aug 11;12(8):e0183104. doi: 10.1371/journal.pone.0183104 (PMC5553772; doi:10.1371/journal.pone.0183104)
Supplement: S1 File — (DOCX) [file pone.0183104.s004.docx]

**SUPPLEMENTARY MATERIAL**

Table 1. Description of all individual errors.

| **Condition** | **Error description** | **Task type** | **Participant recovered from error** |
| --- | --- | --- | --- |
| central | Participants opens VTBI dialog instead of bolus dialog. Participant notices mistake and recovers from it. | Bolus | Yes |
| central | VTBI dialog opened instead of bolus | Bolus | yes |
| central | Participant set VTBI volume to 0.3 ml instead of 3 ml | Setup VTBI | no |
| central | Participant did not set VTBI time to 30 minutes | Setup VTBI | no |
| central | VTBI menu opened instead of bolus menu | Bolus | yes |
| central | VTBI volume set to 0.4 instead of 4 ml | Setup VTBI | no |
| central | Participant did not complete assignment. Pump was restarted, but administration rate was not set to 3.5 ml/h | Restart & change rate | no |
| central | VTBI volume set to 0.3 instead of 3 ml | Replace syringe | no |
| conventional | TIV is not turned off correctly | Stop VBTI | no |
| conventional | TIV is not turned off correctly | Stop VBTI | no |
| conventional | TIV end action should be STOP | Setup VTBI | no |
| conventional | TIV end action should be STOP | Setup VTBI | no |
| conventional | IV rate should be 0.8 ml/h instead of 8 ml/h. Participant does recover | Change rate | yes |
| conventional | TIV menu opened | Stop pump | yes |
| conventional | Pump turned off | Stop pump | no |
| conventional | TIV is not turned off correctly | Stop VBTI | no |
| conventional | TIV is not turned off correctly | Stop VBTI | no |
| conventional | End action should be STOP | Setup VTBI | no |
| conventional | Participant replaced propofol syringe instead of insulin syringe | Replace syringe | yes |
| conventional | Participant tried to do TIV with bolus menu | Setup VTBI | yes |
| conventional | VTBI menu opened instead of change rate | Change rate | yes |
| conventional | Pump stopped | Change rate | yes |
| conventional | Participant stopped bolus halfway and restarts. 3.2 ml infused instead of 3 ml | Bolus | no |
| conventional | Participant set 3 ml TIV instead of 4 ml | Setup VTBI | no |
| conventional | Wrong end action VTBI set. Should be STOP but set to DOORGAAN | Setup VTBI | no |
| conventional | Wrong end action VTBI set. Should be STOP but set to DOORGAAN | Setup VTBI | no |
| conventional | Wrong end action VTBI set | Setup VTBI | no |
| conventional | VTBI should be set to STOP instead of continue | Setup VTBI | no |
| conventional | VTBI was turned off | Setup VTBI | no |
| conventional | Rate of 0.4 ml/h set instead of 4.0 ml/h | Setup VTBI | no |

Table 2. Questionnaire statements, median ratings and Mann Whitney U test results.

|  | **Conventional condition median** | **Central condition median** | **Mann Whitney U** | **P-value** |
| --- | --- | --- | --- | --- |
| 1 This user-interface is easy to use | 5 | 5 | 31.5 | 0.33 |
| 2 This user-interface is aesthetically pleasing | 3.5 | 5 | 4 | 0.00** |
| 3 This user-interface is clear and uncluttered | 4 | 5 | 17 | 0.02* |
| 4 This user-interface works in an intuitive fashion | 4 | 5 | 16.5 | 0.02* |
| 5 It is easy to discriminate between different pumps using this user-interface | 3 | 5 | 7.5 | 0.00** |
| 6 All information I need is available at a glance | 5 | 4 | 36 | 0.65 |
| 7 It is easy to recover from errors | 4 | 4 | 28.5 | 0.25 |
| 8 It is easy to find the functionalities I need | 4 | 5 | 34 | 0.52 |
| 9 It is easy to change an administration rate | 5 | 5 | 40.5 | 1.00 |
| 10 It is easy to see which drug is administered by which pump | 4 | 5 | 30 | 0.29 |
| 11 It is easy to find the remaining volume in a syringe | 5 | 5 | 38.5 | 0.84 |
| 12 It is easy to operate the ‘bolus’ menu | 4 | 5 | 24 | 0.10 |
| 13 It is easy to set up a VTBI (volume to be infused) mode | 4 | 4 | 21.5 | 0.24 |
| 14 It is easy to cancel a VTBI (volume to be infused) mode | 4 | 5 | 18 | 0.06 |
| 15 It is easy to change a syringe | 5 | 4 | 31.5 | 0.36 |
| 16 This user-interface supports me during the task at hand | 5 | 5 | 38.5 | 0.84 |
| 17 This user-interface provides clear feedback to my actions | 4 | 4 | 26.5 | 0.17 |
| 18 I feel in control when operating this user-interface | 5 | 4 | 36 | 0.65 |
| 19 I think that a novice nurse can easily learn to work with this system | 4 | 5 | 31.5 | 0.36 |

**Legend to Table 2.**

**1 indicates the lowest and 5 indicates the highest level of agreement with a statement.**

*** = p < 0.05.**

**** = p < 0.001.**

Task descriptions.

**1 STOP PUMP**

**Conventional control workflow:**

1. Press stop pump button.

**Central control workflow:**

1. Press stop pump button.

**2 RESTART & CHANGE RATE**

**Conventional control workflow:**

1. Press start pump button.
2. Change rate using softkeys. Note: the number of clicks depends on the desired administration rate.
3. Press start pump button.

**Central control workflow:**

1. Press start pump button
2. Press change rate button OR click displayed administration rate.
3. Swipe to desired number and decimal OR use numeric keyboard.
4. Click OK button.

**3 CHANGE RATE**

**Conventional control workflow:**

1. Change rate using softkeys. Note: number of clicks depends on desired flow rate.
2. Press start pump button.

**Central control workflow:**

1. Press change rate button OR click displayed administration rate.
2. Swipe to desired number and decimal OR use numeric keyboard.
3. Click OK button.

**4 BOLUS**

**Conventional control workflow:**

1. Press bolus menu button.
2. Optional: Change bolus rate using softkeys. Note: number of clicks depends on desired flow rate.
3. Hold bolus softkey.
4. Release bolus softkey when desired volume is administered.

**Central control workflow:**

1. Press bolus menu button.
2. Optional: Swipe to desired administration rate.
3. Swipe to desired volume.
4. Click start bolus button.
5. Bolus stops automatically when set volume is administered. Note: an early stop is possible by pressing stop bolus key.

**5 REPLACE SYRINGE**

**Conventional control workflow:**

1. Press stop pump button.
2. Remove syringe.
3. Place new syringe.
4. Press confirm syringe button.
5. Press start pump button.

**Central control workflow:**

1. Click change syringe button. Change syringe dialog opens.
2. Click pause pump button.
3. Remove syringe.
4. Place new syringe.
5. Click “I have changed the syringe on the pump”. Note: this step should be omitted in the future as a placed syringe could be automatically detected.
6. Confirm the syringe on the pump. Note: in the future either step 6 or 7 will be required, not both.
7. Click “I have confirmed the syringe on the pump”. Note: in a future version a confirmed syringe should be automatically detected on the central display.
8. Pump automatically restarts.
9. Click OK to close window.

**Task type: SETUP VTBI**

**Conventional control workflow:**

1. Press VTBI button.
2. Calculate desired volume and rate. Note: this must be done by hand or using a separate calculator.
3. Use softkeys to set number and decimal for desired volume. Note: the number of clicks depends on the desired volume.
4. Press OK button.
5. Use softkeys to select VTBI end action (stop pump, keep vein open protocol or continue infusion).
6. Press OK.
7. Optional: Change rate using softkeys. Note: number of clicks depends on desired flow rate.
8. If step 7 if performed: Press start pump button.

**Central control workflow:**

1. Click VTBI button. VTBI menu opens.
2. Swipe to set desired volume.
3. Optional: click to set desired units (ml, IE, mh, mmol). Note: if drug is known to the system, the appropriate unit is automatically selected).
4. Swipe to set end action (stop pump, keep vein open protocol or continue infusion).
5. Optional: click to infuse at current rate or within a time interval.
6. If in step 5 user selected a time interval: swipe to set hours and minutes. Note: the required administration rate will automatically be calculated and displayed.
7. Click start VTBI button.

**Task type: STOP VTBI**

**Conventional control workflow:**

1. Press VTBI button.
2. Use softkeys to set remaining volume to 0.0 ml. Note: the number of clicks depends on the remaining volume.
3. Press OK button.

**Central control workflow:**

1. Click VTBI button. VTBI menu opens.
2. Click cancel VBTI button.
3. Click confirm button.
